# Large mesenchymal cystic and chondroid pulmonary hamartoma mimicking lung cancer: Case report

**DOI:** 10.1186/s13019-023-02394-z

**Published:** 2023-10-10

**Authors:** Seha Ahn, Heejin Lee, Joon Kyu Kang, In Sub Kim, Youngkyu Moon, Jung Suk Choi, Si Young Choi

**Affiliations:** Department of Thoracic & Cardiovascular Surgery. Eunpyeong St. Mary’s Hospital, College of Medicine, The Catholic University of Korea, 1021, Tongil-ro, Eunpyeong-gu, Seoul, 03312 Republic of Korea

**Keywords:** Cystic hamartoma, Chondroid hamartoma, Mimicking, Lung cancer

## Abstract

Pulmonary hamartoma is the most commonly resected benign neoplasm of lung. The mesenchymal cystic subtype is a rare and often bilaterally occurring variant composed of multiple cysts and nodules. Herein, we present an asymptomatic 70-year-old woman with a large and mostly cystic growth of right hilar region. Computed tomography of the chest and fluorodeoxyglucose positron emission tomography/computed tomography imaging traced its origins to right middle lobe. Overall features suggested primary lung cancer or perhaps other cystic lung disease.

Because transbronchial lung biopsy failed to establish a histologic diagnosis, right middle lobectomy was undertaken by video-assisted thoracoscopic surgery. The gross surgical specimen harbored a single and sizeable (8.0 × 4.0 cm) cystic lesion containing multiple yellow-white nodules. A diagnosis of mesenchymal cystic and chondroid hamartoma was ultimately rendered. This particular case is noteworthy, given the initial clinical resemblance to primary lung cancer.

## Introduction

Pulmonary hamartoma is the most commonly resected benign lung tumor, affecting 0.025–0.032% of the adult population [1]. Typically, such growths replicate normal lung constituents, including fat, mucinous fibrous connective tissue, ciliated epithelium, and respiratory epithelial-lined adenoid structures (2). Mesenchymal cystic hamartoma (MCH) is a rare variant first reported in 1986. It is largely comprised of primitive mesenchymal cells that form multiple cysts and nodules, often arising bilaterally. These tumors grow slowly and tend to become cystic at diameters > 1 cm [2].

The elderly woman we describe was initially thought to have primary lung cancer with cystic change. Instead, a mesenchymal cystic and chondroid hamartoma was confirmed through surgical resection.

## Case presentation

**Fig. 1:**
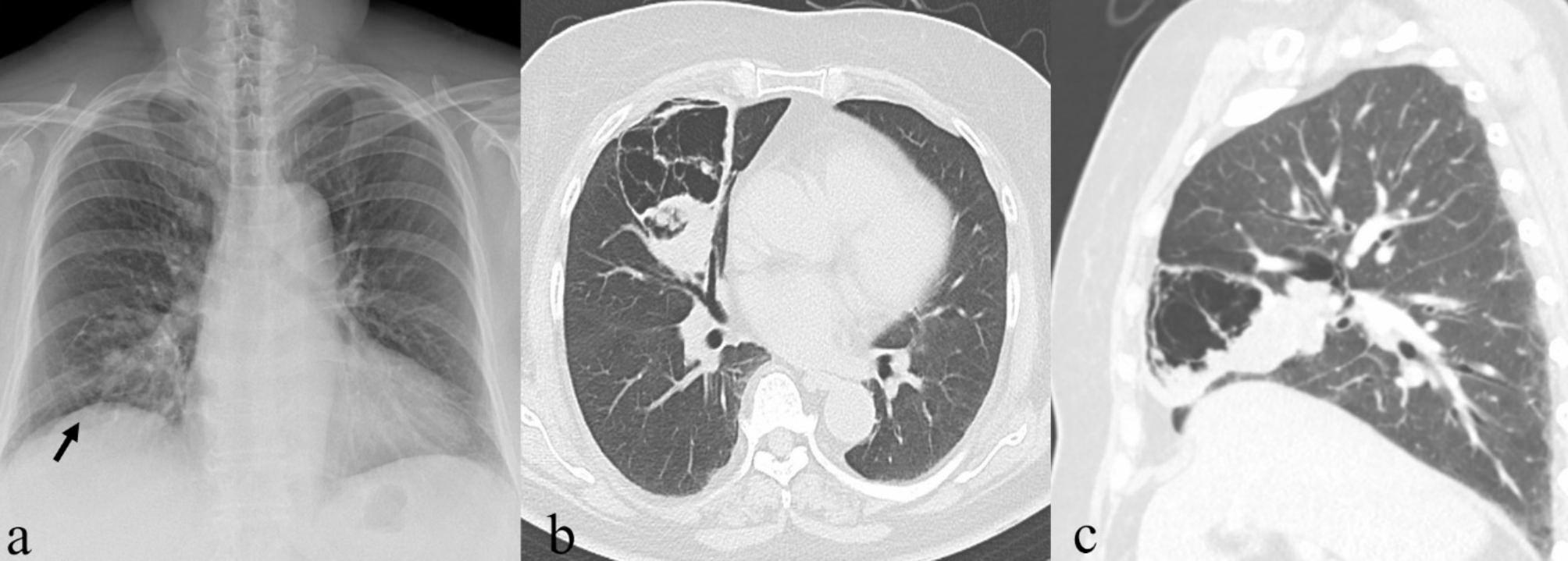
Preoperative imaging studies: (A) cystic area (black arrow) and ground-glass opacity of right hilar region detected by chext X-ray; and (B, C) computed tomography of chest (axial and sagittal views) demonstrating large cystic area (8.8 × 6.1 cm) and solid growth (4.9 cm) of right middle lobe

An outpatient, a 70-year-old woman with no history of smoking, presented our Department of General Surgery with bilateral lower leg edema. Historically, a right lung nodule encountered 15 years prior had received no subsequent follow-up. The patient has a medical history that includes hypertension, diabetes mellitus, and hyperlipidemia. In terms of past surgeries, she underwent total knee replacement surgery for both knees, with the right side operated on 5 months ago and the left side 11 years ago, both performed at a different institution. Chest X-ray now disclosed a cystic area and a ground-glass opacity of the right hilar region (Fig. 1A). Computed tomography (CT) of the chest localized both lesions (cystic: 8.8 × 6.1 cm; solid: 4.9 cm) to the right middle lobe (RML) (Fig. 1B, C), suggesting primary lung cancer with cystic change. She was referred to our Pulmonary Department for further evaluation.

The patient was asymptomatic on physical exam. Her preoperative pulmonary function tests and echocardiogram results were within normal limits. In the absence of overt endobronchial pathology during bronchoscopy, a transbronchial lung biopsy and lavage fluid were routinely obtained. Fluorodeoxyglucose positron emission tomography/computed tomography (^18^ F-FDG PET/CT) showed a large cystic area of RML, with mild FDG uptake. There was also spotty FDG uptake at right lower paratracheal and subcarinal locations and involving both hilar and peribronchial regions. The interpretation of ^18^ F-FDG PET/CT images in the right lower paratracheal and subcarinal regions indicated reactive lymph nodes, leading to a diminished suspicion of lymph node metastasis. Despite favoring a chronic inflammatory process with cyst formation, efforts to exclude malignancy were warranted. The transbronchial lung biopsy was not helpful (respiratory epithelium only), and the inordinate risk of pneumothorax precluded CT-guided percutaneous cutting needle biopsy (PCNB). Diagnosis and treatment were then entrusted to our Department of Thoracic and Cardiovascular Surgery.

Right middle lobectomy was eventually performed by video-assisted thoracoscopic surgery (VATS). The patient was placed in left lateral decubitus position, using a double-lumen endotracheal tube for single-lung ventilation. An incision (4.0 cm) was made in anterior axillary line at fifth intercostal space (ICS), followed by two additional 10-mm incisions of mid-axillary and posterior axillary lines at seventh ICS. Upon inspection, a thick-walled cavitary lesion of RML was noted. The resected specimen was collected by Endo Catch bag (Medtronic, Minneapolis, MN, USA) to retrieve via working port for frozen section diagnosis. Tissue assessed during mediastinal lymphadenectomy appeared benign, suggestive of hamartoma, so no further procedures were done. Operative time was 195 min, and the duration of anesthesia was 240 min. The estimated blood loss was 100 cc.

**Fig. 2:**
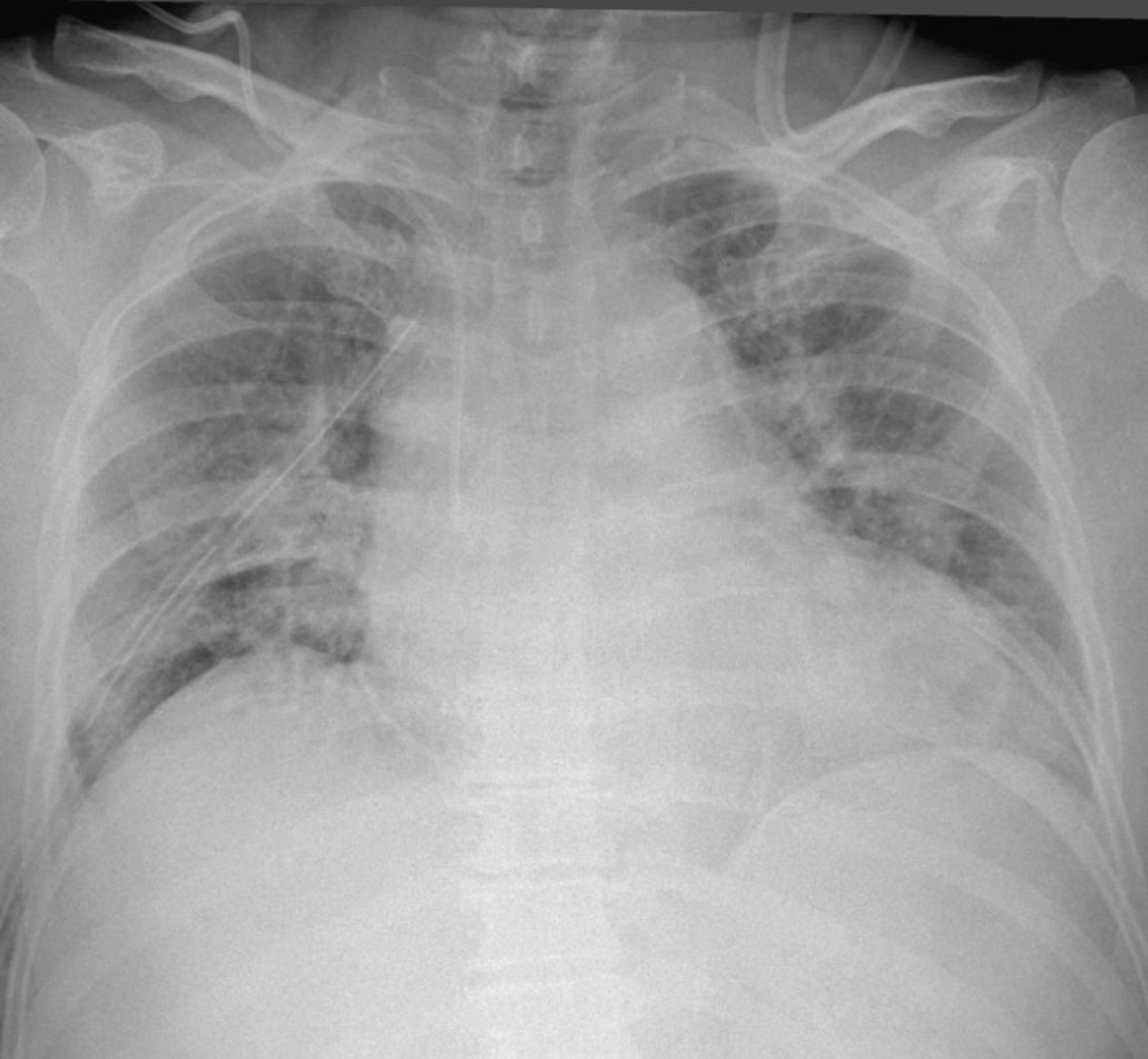
Postoperative chest X-ray (upright, anteroposterior) clear in recovery room

**Fig. 3:**
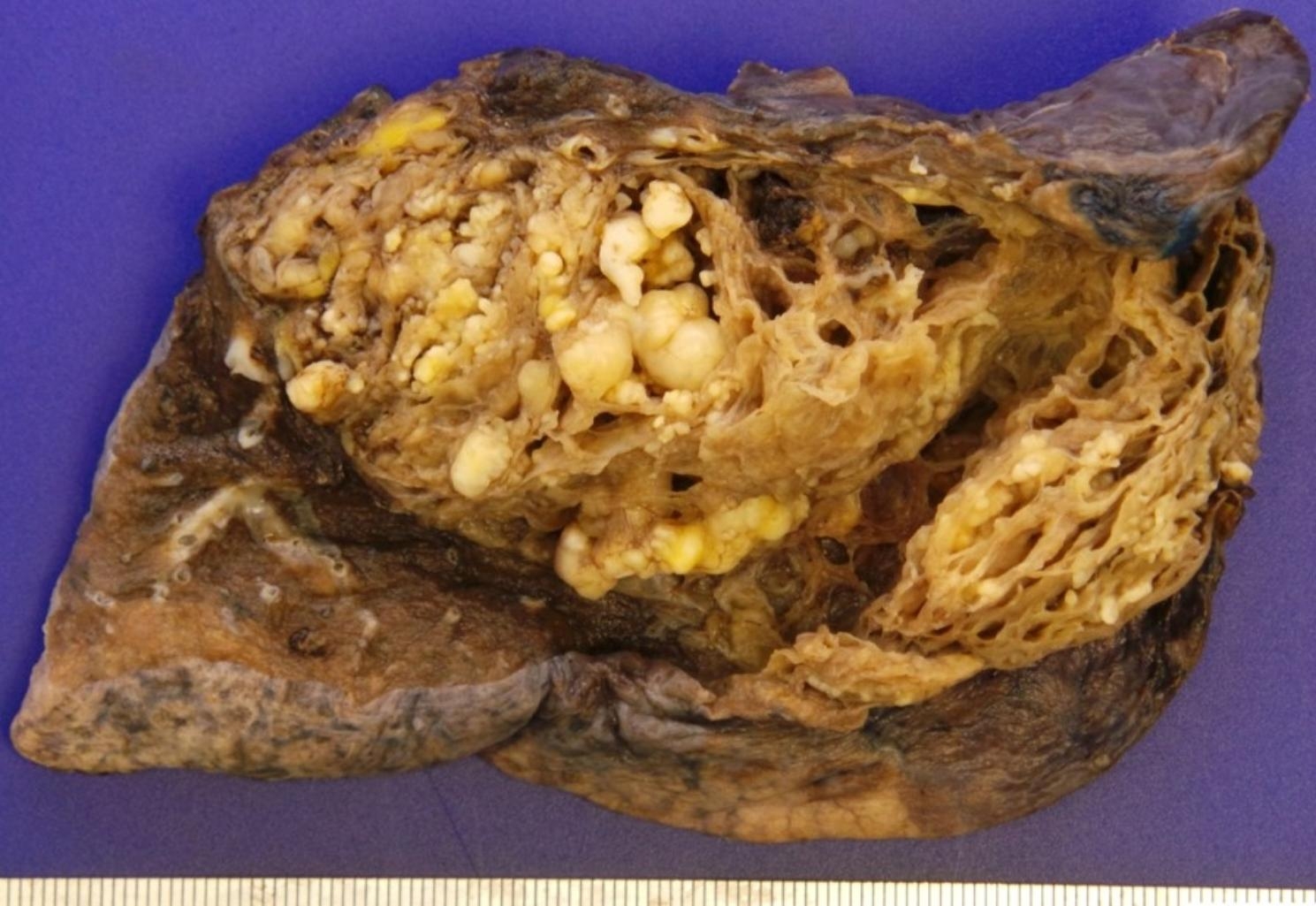
Gross appearance of surgical specimen, demonstrating single, large cystic growth (8.0 × 4.0 cm) with multiple yellow-white nodules

An upright anteroposterior chest X-ray obtained in the recovery room (20 min after procedural completion) was clear (Fig. 2). As depicted radiographically, the resected lung specimen (Fig. 3) grossly displayed a single large cystic mass (13.0 × 9.0 × 4.5 cm) containing multiple yellow-white nodules. A mesenchymal cystic and chondroid hamartoma was confirmed by the final pathology report. Additionally, the resection margin was clear, and the pleural surface exhibited a smooth and glistening appearance. Furthermore, the paratracheal lymph node and hilar lymph node were negative, indicating the absence of cancer involvement. The chest tube was removed on postoperative Day 5, and no complications were observed in the patient. However, to ensure better pain control, the patient was discharged on postoperative Day 9. Her clinical course thereafter has been uneventful for 36 months.

## Discussion

In this particular instance, the initially suspected primary lung cancer (with cystic change) proved to be a rare variant of pulmonary hamartoma. Various subtypes are categorized by the major component: cartilage (chondroid hamartoma, most common), smooth muscle, or connective tissue [1]. In CT studies, hamartomas typically present as single, well-defined nodules that are smooth-surfaced and round or lobulated. The radiographic hallmark of chondroid hamartoma is a fatty density with popcorn-like calcifications [1, 3].

MCH is a rare and often bilateral growth in which largely primitive mesenchymal cells proliferate, forming multiple cysts and nodules [2]. Hemoptysis, pneumothorax, hemothorax, pleuritic chest pain, or dyspnea, may or may not accompany these tumors [4, 5]. Discovery by X-ray in our asymptomatic patient was incidental. The differential diagnosis includes other benign and radiographically similar diseases (i.e., pleuropulmonary blastoma, cystic adenomatoid malformation, and lymphangiomyomatosis), with malignant transformation as a remote possibility [4, 6, 7].

The metabolic activity observed on ^18^ F-FDG PET/CT for pulmonary hamartoma typically appears absent or low, similar to other benign lung lesion [8]. Several factors contribute to the diagnosis of pulmonary hamartoma using 18 F-FDG PET/CT, such as the presence of a single lesion, a low maximum standardized uptake value (SUVmax) of the lesion (falling below the internationally recognized cut-off value of 2.5), and the absence of any other coexisting malignant tumors [8–10]. At our institution, outpatient follow-up is performed for suspected cases of typical hamartoma. In cases where a large mass is present and distinguishing it from primary lung cancer is challenging, 18 F-FDG PET/CT scans are conducted. If deemed necessary, PCNB or surgical resection is performed for further evaluation. In our specific case, a large solid tumor with a cystic component was a rare variant of pulmonary hamartoma, and the size of the lesion did not correlate with typical hamartoma characteristics. Furthermore, it was essential to differentiate the patient’s large solid tumor with a cystic component from primary lung cancer. Despite the presence of mild FDG uptake on the 18 F-FDG PET/CT scan, accurate discrimination between these two conditions was of utmost importance. CT-guided PCNB was not a viable option in the wake of an unproductive transbronchial lung biopsy. Cystic lesions carry a high risk of pneumothorax and obfuscate needle localization. Furthermore, withdrawal of fluid was likely, making accurate diagnosis difficult [11]. We finally settled on a surgical approach for diagnosis and treatment. Following RML resection, the patient has remained recurrence-free for 36 months.

## Conclusion

This 70-year-old woman was initially thought to have primary lung cancer with cystic change. Tissue examination later revealed a rare variant of pulmonary hamartoma, specifically a mesenchymal cystic and chondroid subtype.

## Data Availability

The data underlying this article will be shared on reasonable request to the corresponding author.

## Abbreviations

MCH: Mesenchymal cystic hamartoma
CT: Computed tomography
RML: Right middle lobe
^18^F-FDG PET/CT: Fluorodeoxyglucose positron emission tomography/computed tomography
PCNB: percutaneous cutting needle biopsy
VATS: Video-assisted thoracoscopic surgery
ICS: Intercostal space
SUV: Standardized uptake value
